# The DNA structure and sequence preferences of WRN underlie its function in telomeric recombination events

**DOI:** 10.1038/ncomms9331

**Published:** 2015-09-30

**Authors:** Deanna N. Edwards, Amrita Machwe, Li Chen, Vilhelm A. Bohr, David K. Orren

**Affiliations:** 1 Department of Toxicology and Cancer Biology, University of Kentucky College of Medicine, Lexington, Kentucky 40536, USA; 2 Markey Cancer Center, University of Kentucky College of Medicine, Lexington, Kentucky 40536, USA; 3 Department of Cancer Biostatistics, University of Kentucky College of Public Health, Lexington, Kentucky 40536, USA; 4 Laboratory of Molecular Gerontology, National Institute on Aging, National Institutes of Health, Baltimore, Maryland 21224, USA

## Abstract

Telomeric abnormalities caused by loss of function of the RecQ helicase WRN are linked to the multiple premature ageing phenotypes that characterize Werner syndrome. Here we examine WRN’s role in telomeric maintenance, by comparing its action on a variety of DNA structures without or with telomeric sequences. Our results show that WRN clearly prefers to act on strand invasion intermediates in a manner that favours strand invasion and exchange. Moreover, WRN unwinding of these recombination structures is further enhanced when the invading strand contains at least three G-rich single-stranded telomeric repeats. These selectivities are most pronounced at NaCl concentrations within the reported intranuclear monovalent cation concentration range, and are partly conferred by WRN’s C-terminal region. Importantly, WRN’s specificity for the G-rich telomeric sequence within this precise structural context is particularly relevant to telomere metabolism and strongly suggests a physiological role in telomeric recombination processes, including T-loop dynamics.

## Abstract (web summary)

The loss of WRN helicase leads to abnormalities at chromosome ends and is associated with premature ageing phenotypes characteristic of Werner syndrome. Here the authors show that WRN acts in a structure- and sequence-specific manner on recombination intermediates relevant to telomere maintenance.

## Graphical abstract

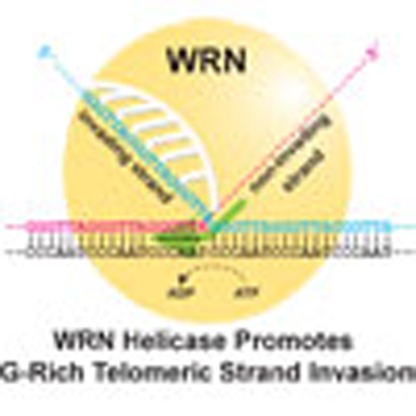

Human chromosomes are capped by telomeres containing noncoding, repetitive TTAGGG/AATCCC duplex DNA sequences, ending with 3′ overhangs of the G-rich strand. In dividing somatic cells, telomeric regions become shortened due to the end-replication problem, stochastic deletion events and insufficient activity of telomerase, the reverse transcriptase present in germ, stem and most cancer cells that adds back a telomeric sequence^1^,^2^,^3^,^4^. Indeed, telomere length is associated with cellular replicative capacity and correlations exist between donor age and *in vitro* replicative potential^5^,^6^, suggesting telomere shortening and dysfunction contributes to ageing. Dysfunctional telomeres are revealed as double-strand breaks that initiate an ATM- and p53-dependent DNA damage response, whereas functional telomeres suppress this response as well as telomeric fusions, thus protecting both telomeric and genomic integrity^7^,^8^,^9^. Telomeric protection involves (recombination-like) invasion and sequestration of G-rich 3′ overhangs within telomeric duplex regions, forming so-called T-loops^10^,^11^,^12^. A group of proteins collectively termed shelterin, which in humans includes TRF1, TRF2, POT1, TIN2, RAP1 and TPP1, interact specifically with telomeres and regulate their structure and function^9^,^13^.

Several human diseases are associated with telomere instability including Werner syndrome (WS), an autosomal recessive disorder characterized by premature emergence of numerous ageing phenotypes that include cataracts, atherosclerosis and increased cancer susceptibility^14^,^15^,^16^. Amazingly, these multiple ageing features result from defects in a single gene product, WRN^17^. Forced expression of telomerase prevents premature cellular senescence that occurs in primary WS fibroblasts^18^, strongly suggesting that telomeric defects cause this accelerated senescence. Moreover, WRN-deficient cells exhibit stochastic telomere loss^19^,^20^ and other telomere-related abnormalities^21^,^22^. WRN associates with telomeres during the S-phase^20^,^23^ and functionally interacts with shelterin components TRF2 and POT1 (refs 23, 24, 25, 26, 27, 28). Most importantly, Wrn deficiency specifically led to telomeric defects and premature ageing features in late-generation, telomerase-deficient mice with ‘pre-shortened’ telomeres^29^,^30^. Although this evidence indicates a telomeric role for WRN, its precise molecular function remains unclear.

As a RecQ family member, WRN possesses 3′–5′ helicase and strand-annealing activities along with 3′–5′ exonuclease activity^31^,^32^,^33^,^34^. WRN action appears most robust on DNA structures reflecting replication, repair and recombination intermediates^35^,^36^,^37^,^38^,^39^. Consistent with a possible role in homologous recombination (HR) processes, WRN also coordinates its helicase and strand-annealing activities to catalyse strand exchange^33^. To investigate WRN’s potential role in telomeric recombination, here we examine WRN function on strand invasion intermediates without and with telomeric sequences. Our results demonstrate that WRN preferentially acts on these recombination intermediates and with a directionality promoting further strand invasion. Importantly, this activity is further enhanced by the presence of single-stranded, unstructured G-rich telomeric sequence along the invading strand, a structural context precisely relevant to telomeric HR and T-loop dynamics. WRN’s C-terminal region, downstream of its helicase domain, contributes to these structure and sequence preferences. Our results strongly suggest that WRN specifically acts in telomeric HR processes possibly including T-loop formation.

## Results

### Preferential action of WRN on strand invasion intermediates

**Figure 1:**
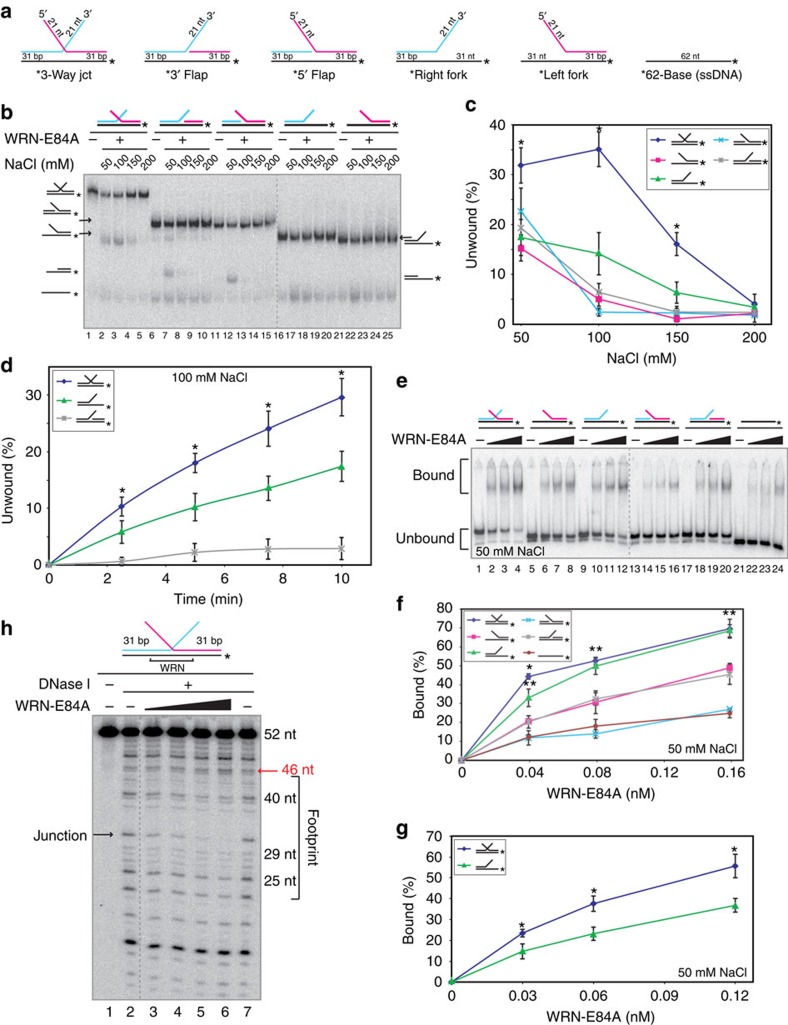
WRN preferentially acts on strand invasion intermediates. (**a**) Basic 3-way jct and related substrates, with 5′- and 3′-flap strand variations in magenta and turquoise, respectively. (**b**) Helicase assays were performed for 10 min using WRN-E84A (0.45 nM) on *3-way jct, *3′ Flap, *5′ Flap, *Right Fork or *Left Fork substrates (0.2 nM each) in 50–200 mM NaCl as specified; substrate and product migrations are noted. (**c**) Percentages (mean±s.e.m. of four experiments, as in **a**) of each substrate unwound are plotted versus NaCl concentration. (**d**) Helicase assays on *3-way jct, *Right Fork and *3′ Flap substrates (0.2 nM each) in 100 mM NaCl were performed for 0–10 min and analysed as in **a**. Percent unwinding (mean±s.e.m. of five experiments) for each substrate is plotted versus time. (**e**) EMSA with WRN-E84A (0, 0.04, 0.08 or 0.15 nM) and *3-way jct, *Left Fork, *Right Fork, *5′ Flap, *3′ Flap,or *62-base substrate (0.1 nM each) in 50 mM NaCl for 10 min at 37 °C. After adding one-sixth volume of 30% glycerol, samples were analysed by native PAGE at 25 °C. (**f**) Percent (mean±s.e.m. of four experiments, as in **e**) of substrate bound plotted versus WRN concentration. (**g**) WRN-E84A (0, 0.03, 0.06 or 0.12 nM) incubated with *3-way jct or *Right Fork (0.1 nM each) was analysed as in **e**, except 0.25% BPB and 0.25% XC were included with added glycerol. Binding (mean±s.e.m. of five experiments) was calculated as in **f**. (**c**,**d**,**f**,**g**) Asterisks indicate significant differences (*P*<0.035, 0.0066, 0.019 and 0.0024) between values for *3-way jct compared with analogous data points for each other substrate, while double asterisks in **f** indicate significance (*P*<0.0044) between both *3-way jct and *Right Fork compared with remaining substrates; all *P* values were determined using two-tailed unpaired Student’s *t*-tests. (**h**) Reactions containing WRN-E84A(0, 0.72, 1.4, 2.9 or 5.8 nM) and *3-way jct (2.1 nM) were analysed by DNase I footprinting. Fragment sizes (right) and locations of the junction (black arrow), hypersensitive site (red arrow) and WRN footprint (bracket) are indicated.

Since WRN catalyses strand exchange and has affinity for multistranded recombination intermediates^33^,^35^,^36^,^37^,^38^,^39^ potentially relevant to its telomeric function, we first investigated WRN activities on three-way junction substrates reflecting strand invasion HR intermediates that are also key structural features of T-loops. Our initial experiments compared three-way junction substrates containing random (non-telomeric) sequences with substrates lacking certain structural elements (Fig. 1a). These and other static three-way junction substrates (Supplementary Tables 1 and 2 specify oligo and substrate composition) used hereafter contained a common labelled oligomer (*62-base) to facilitate comparison based on radioactivity. The basic three-way junction substrate (*3-way jct) contained 5′ and 3′ flaps of 21 nt each and two 31-bp regions with similar nucleotide content to avoid unwinding bias for either duplex. On the basis of parallels with HR intermediates, we often refer to 5′ and 3′ single-stranded flap strands as invading and non-invading strands, respectively. Other DNA substrates (Fig. 1a) structurally related to *3-way jct included (1) *Left Fork, lacking the entire non-invading strand, (2) *Right Fork, lacking the entire invading strand, (3) *5′ Flap, lacking only the single-stranded 3′ flap and (4) *3′ Flap, lacking only the single-stranded 5′ flap. Since intranuclear monovalent cation concentration is reported to be as high as 250 mM (refs 40, 41, 42), effects of NaCl concentration were examined here and in many subsequent experiments addressing WRN’s DNA structure and sequence preferences. Adenosine triphosphate (ATP)-dependent WRN unwinding on these substrates was revealed by appearance of faster-migrating products after native polyacrylamide gel electrophoresis (PAGE) (Fig. 1b). Exonuclease-deficient WRN-E84A, hereafter simply identified as WRN, was used to prevent substrate digestion and thereby allow straightforward evaluation of helicase activity, at sub-saturating concentrations for *3-way jct unwinding. These experiments (Fig. 1b,c) show an obvious, significant preference for WRN unwinding of *3-way jct between 50 and 150 mM NaCl compared with other substrates that was most prominent at 100 mM NaCl. For all data derived from multiple experiments (here and subsequently), error bars indicate s.e.m. and *P* values are calculated using two-tailed unpaired Student’s *t*-tests. A kinetic analysis at 100 mM NaCl with *3-way jct, *3′ Flap and *Right Fork substrates demonstrated a similar and statistically significant preference for unwinding *3-way jct within 2.5 min (Fig. 1d), confirming that these results reflect structure-specific WRN unwinding and not differential annealing. Clearly, WRN preferentially unwinds 3-way junction structures resembling strand invasion intermediates; importantly, this preference is most pronounced at 100–150 mM NaCl.

To determine whether this structure preference was due to higher DNA-binding affinity, electrophoretic mobility shift assays (EMSAs) were used to examine WRN binding to these same substrates, as well as single-stranded DNA. While discrete, concentration-dependent WRN–DNA complexes were observed with all substrates, differences in WRN affinity between substrates were evident (Fig. 1e). The general hierarchy of WRN binding was: *3-way jct and *Right Fork>*Left Fork and *3′ Flap>*5′ Flap and *62-base (Fig. 1f). More specifically, WRN binding to *3-way jct was significantly better than all other substrates at the lowest WRN concentration, while binding to both *3-way jct and *Right Fork was significantly better than other substrates for all WRN concentrations (Fig. 1f). Although this pattern of structural discrimination showed similarities to our unwinding results, we further interrogated binding to *3-way jct and *Right Fork substrates, exploiting our knowledge that WRN–DNA binding is negatively impacted by xylene cyanol (XC) and bromophenol blue (BPB) dyes (typically added to assist sample loading and tracking). Under these more stringent conditions, binding was significantly higher to *3-way jct compared with *Right Fork across WRN concentration (Fig. 1g). These results indicate that WRN binds with higher affinity and stability to 3-way junctions than to other structures, and suggest that enhanced binding is at least partly responsible for WRN’s increased unwinding efficiency on model strand invasion intermediates.

In addition, we used DNase I footprinting to pinpoint where WRN binds relative to the duplex regions of *3-way jct substrate (Fig. 1h). Without WRN, DNase I produced a characteristic DNA fragment ladder, including a prominent 31-nt band essentially at the junction point and weak cutting immediately 3′ to the junction (Fig. 1h, lanes 2 and 7). With increasing WRN, the 31-nt band progressively disappeared, accompanied by more modest decreases of the 22–40-nt bands (lanes 3–6). In contrast, a 46-nt fragment was moderately increased, especially at the highest WRN concentration, indicating a mild hypersensitive site (Fig. 1h, lane 6) that likely highlights the periphery of WRN bound to this duplex region. This evidence indicates that WRN protects an 18–23-bp region centred almost symmetrically around the three-way junction, a binding mode presumably relevant to substrate unwinding.

### WRN unwinds 3′ flap strands of strand invasion structures

**Figure 2:**
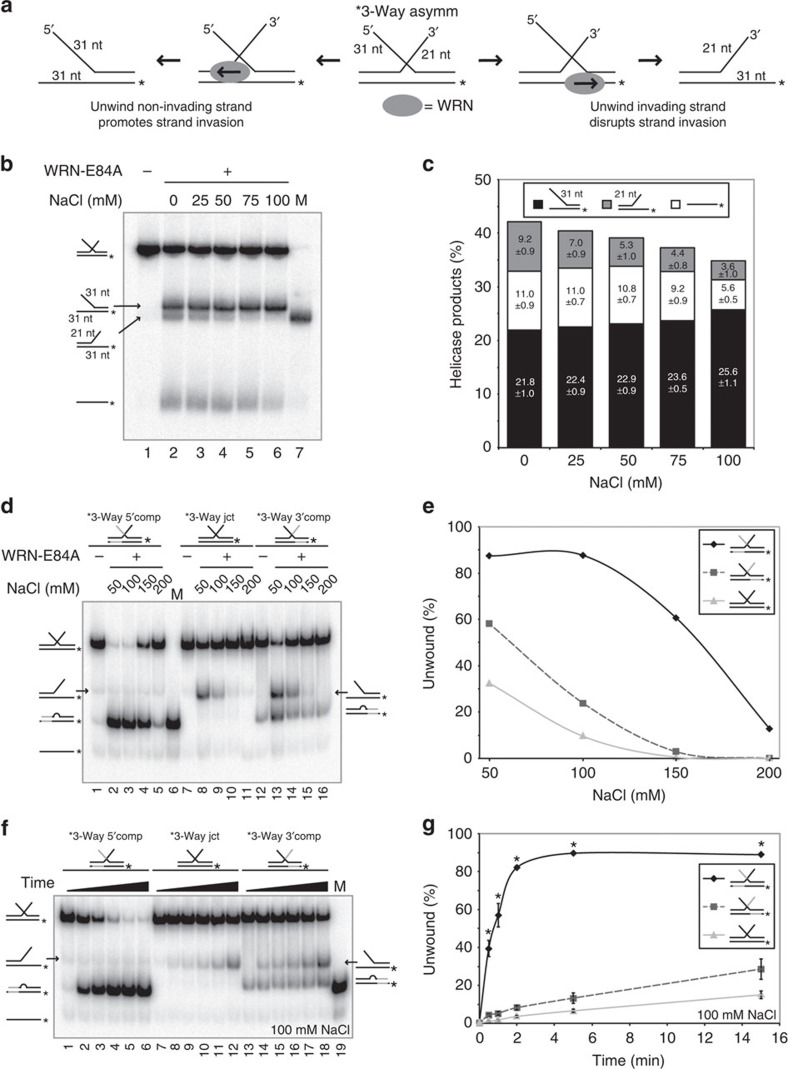
WRN predominantly displaces non-invading strands of strand invasion intermediates. (**a**) Experimental design to determine WRN unwinding directionality on *3-way asymm substrate, showing different two-stranded forks generated by displacing non-invading/3′ flap strand (left) or invading/5′ flap strand (right), promoting or disrupting, respectively, typical strand invasion intermediates. (**b**) Helicase assays performed as previously on WRN-E84A (2.5 nM) incubated 15 min with *3-way asymm (0.2 nM) in 0–100 mM NaCl, including marker (M) for a two-stranded fork with two 21-nt flaps (lane 7). Migrations of substrate and products are indicated. (**c**) Stacked bar graph showing unwinding product amounts (mean±s.e.m. of three experiments, as in **b**) at each NaCl concentration. (**d**) Helicase assays were performed on WRN-E84A (0.48 nM) incubated 15 min with *3-way 5′comp, *3-way jct or *3-way 3′comp substrate (0.2 nM each) in 50–200 mM NaCl as indicated, including a marker (M) for bubble product (lane 6). Migrations of substrates and products are shown. (**e**) Percent unwinding for assays in **d** with *3-way 5′comp (diamonds), *3-way jct (triangles) or *3-way 3′comp (squares) substrate plotted at each NaCl concentration. (**f**) Helicase assays performed as in **d**, except specifically in 100 mM NaCl for 0, 0.5, 1, 2, 5 or 15 min and including marker (M) for bubble product (lane 19). (**g**) Unwinding (mean±s.e.m. of three experiments, performed as in **f**) was plotted versus time, with symbols as in **e**. Asterisks denote statistical significance (*P*<0.0004, determined using two-tailed unpaired Student’s *t*-tests) of values for *3-way 5′comp substrate compared with analogous data points for other substrates.

The directionality of WRN action on three-way junction structures defines whether WRN promotes or disrupts strand invasion. Results above (Fig. 1b, lanes 2–4) indicated that WRN generally unwinds one strand of *3-way jct, yielding a two-stranded fork. However, the two fork products formed from unwinding this symmetric substrate migrate very similarly (Fig. 1b, lanes 16 and 21). To clarify WRN unwinding directionality on these structures, another three-way junction substrate, *3-way asymm, with a longer (31 nt) 5′ flap was designed so that the fork produced by 3′-flap strand unwinding should migrate slower than the fork generated by 5′-flap strand displacement (Fig. 2a). Unwinding of *3-way asymm substrate was assessed in reactions containing 0–100 mM NaCl. Without NaCl, WRN generated both forks and some single-stranded product (Fig. 2b, lane 2); importantly, the weaker fork product migrated slightly faster and exactly like the fork with two shorter (21 nt) arms (Fig. 2b, lane 7). With increasing NaCl, this faster-migrating fork product resulting from 5′-flap strand displacement progressively disappeared, while the more abundant, slower-migrating fork seemed unaffected (Fig. 2b, lanes 3–6). Quantification of multiple experiments (Fig. 2c) confirmed that generation of the slower-migrating fork stayed relatively constant or even increased slightly with increasing NaCl concentration (21.8–25.6%), while amounts of the faster-migrating fork dropped from 9.2% without NaCl to 3.6% at 100 mM NaCl; single-stranded product also declined with increasing NaCl concentration, reflecting reduced secondary unwinding of one or both forks. Clearly, WRN preferentially unwinds the 3′ flap strand of three-way junction structures, and this preference becomes nearly absolute at 100 mM NaCl. Interestingly, this preferential, salt-resistant unwinding of the 3′ flap strand depended upon the presence of a 5′ flap, as WRN unwinding of *3′ Flap substrate lacking this 5′ flap showed primarily the opposite directionality without NaCl and was drastically inhibited as NaCl concentration increased (Supplementary Fig. 1a,b). Notably, a different substrate (*3-way 5′ 5 nt) containing a short (5 nt) 5′ flap restored the unwinding directionality pattern observed with the *3-way asymm substrate that persisted even at 100 mM NaCl (Supplementary Fig. 1a,b). Together, these results demonstrate that strand invasion intermediates are high-affinity structures for WRN, which exclusively displaces the 3′ flap (non-invading) strand at Na^+^ levels within the reported intranuclear monovalent cation concentration range^40^,^41^,^42^. Importantly, this directionality promotes further strand invasion and exchange. Intriguingly, the mere presence of a single-stranded 5′ flap markedly influences WRN-mediated unwinding, suggesting that WRN senses the invading strand within strand invasion intermediates even though it displaces the non-invading strand.

### Homology boosts WRN unwinding of strand invasion structures

Since strand exchange normally occurs between homologous DNA segments, we also examined the effect of sequence complementarity on WRN action. Thus, we designed new three-way junction substrates, *3-way 5′comp and *3-way 3′comp, with 5′ and 3′ single-stranded flaps, respectively, for which the distal 16 nt were complementary (to corresponding duplex regions), while 5 nt of non-complementarity adjacent to the junction prevented spontaneous branch migration (Fig. 2d,e). Original *3-way jct substrate lacking complementarity served as comparative control and, as previously, WRN unwound this substrate to produce a two-stranded fork (Fig. 2d, lanes 8–11). However, reactions on partly complementary three-way junction substrates showed marked differences with the control and each other. On *3-way 3′comp substrate possessing 3′-flap homology (Fig. 2d, lanes 12–16), WRN generated two products of similar intensity, indicating some 5′-flap strand unwinding to yield a faster-migrating, 5-bp bubble product. Although the sum of these products was greater than for *3-way jct, both products were reduced as NaCl concentration increased and unwinding was essentially abolished at 150 mM NaCl (Fig. 2d,e); note the residual levels of bubble observed at 150–200 mM NaCl were present in the substrate preparation without WRN (Fig. 2d, lane 12). In contrast, on *3-way 5′comp substrate with 5′ flap homology (Fig. 2d, lanes 1–5), WRN exclusively generated 5-nt bubble products by displacing the 3′ flap strand, the directionality observed with *3-way jct asymm (Fig. 2b,c), with more efficient unwinding across NaCl concentration (Fig. 2e). Kinetic experiments performed at 100 mM NaCl showed that reaction rates on this substrate were very significantly enhanced compared with other substrates at each time point, with unwinding at 39.5% after 30 s and near completion (89.6%) after 5 min (Fig. 2f,g). These results demonstrate that sequence homology specifically on the 5′ flap greatly stimulates unwinding of the 3′ flap strand and its salt resistance on three-way junction substrates, again emphasizing the importance of this structural feature for WRN’s function in promoting further strand invasion and exchange.

### Effect of telomeric sequence and its placement on WRN action

**Figure 3:**
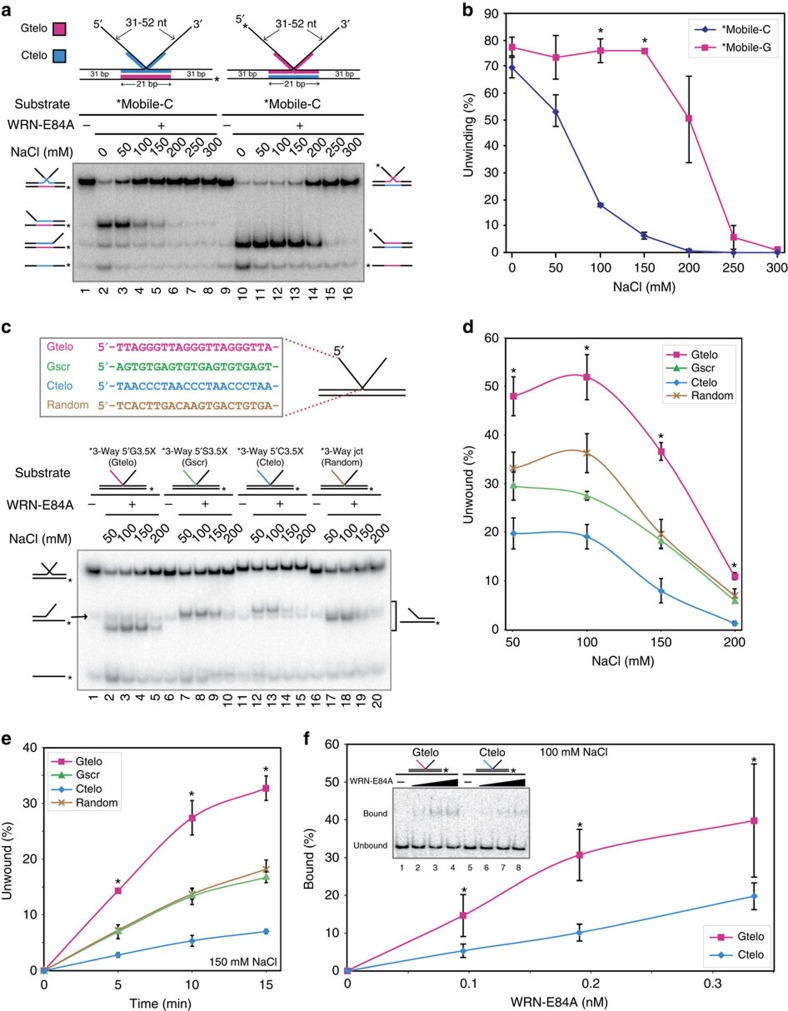
G-rich telomeric sequence specifically on the invading strand of strand invasion intermediates mediates heightened WRN unwinding. (**a**) *Mobile-C and *Mobile-G substrate structures with positions of G-rich and C-rich telomeric sequences in magenta and blue, respectively. Helicase assays with *Mobile-C and *Mobile-G (0.2 nM each) and WRN-E84A (1.2 nM) were performed for 15 min in 1 mM MnCl_2_ and 0–300 mM NaCl as indicated. Migrations of substrates and products are denoted. (**b**) Percent unwinding (mean+s.e.m. of two experiments, as in **a**) of *Mobile-C (blue diamonds) or *Mobile-G (magenta squares) to fork and single-stranded products was calculated and plotted versus NaCl concentration. (**c**) Helicase assays were performed for 15 min on *3-way 5′G3.5X (Gtelo), *3-way 5′S3.5X (Gscr), *3-way 5′C3.5X (Ctelo) or *3-way jct (Random) substrate (0.2 nM each) using WRN-E84A (0.24 nM) in 50–200 mM NaCl as indicated. 5′ flap sequences for each substrate are specified and colour-coded in relation to **d**–**f** (box). (**d**) Percentage of substrate unwound (mean±s.e.m. of four experiments, as in **c**) plotted versus NaCl concentration. (**e**) Helicase assays performed using WRN-E84A (0.2 nM) as in **c**, except specifically in 150 mM NaCl for 0–15 min; percent unwinding (mean±s.e.m. of three independent experiments) is plotted versus time. (**f**) Reactions containing *3-way 5′G3.5X or *3-way 5′C3.5X (0.2 nM each) incubated with WRN-E84A (0, 0.10, 0.19 or 0.34 nM) at 4 °C for 10 min in 100 mM NaCl were analysed by EMSA, adding one-sixth volume of 30% glycerol with electrophoresis at 4 °C (inset). Percent of substrate bound (mean±s.e.m. of five experiments) is plotted versus WRN concentration. For **b** and **d**–**f**, asterisks indicate significant differences (*P*<0.0064, 0.025, 0.0044 and 0.027, respectively, determined by two-tailed unpaired Student’s *t*-tests) of values compared with analogous data points from each of the other substrates.

Given WRN’s putative role in telomere maintenance, we next investigated how telomeric sequences within the context of strand invasion intermediates affected unwinding. We initially constructed three-way junction substrates, designated *Mobile-G or *Mobile-C, that contained G-rich (TTAGGG) or C-rich (AATCCC) telomeric sequences, respectively, proximal to the junction on the 5′ (invading) and 3′ (non-invading) flaps as well as homologous segments on their duplex regions, permitting spontaneous but limited movement of the junction (Fig. 3a). Importantly, placement of telomeric sequences in *Mobile G reflects their position in HR intermediates formed by invasion of telomeric G-rich 3′ overhangs, as also occurs in T-loops. WRN unwinding of these substrates was examined from 0 to 250 mM NaCl. Similar to results with static three-way junction substrates, WRN predominantly displaces 3′ flap strands from both substrates, although minor 5′-flap strand unwinding is observed, particularly without NaCl (Fig. 3a); note that, 5′-flap strand unwinding of *Mobile-G substrate generates single-stranded products (Fig. 3a, lane 10), dictated by the radiolabel’s position. Strikingly, differences in WRN-mediated unwinding of *Mobile-C and *Mobile-G substrates are clearly evident as NaCl concentration increases (Fig. 3a,b). While unwinding of *Mobile-C substrate drops markedly to 18.7 and 7.6% at 100 and 150 mM NaCl, respectively, unwinding of the 3′ flap strand of the *Mobile-G substrate persists at around 80% up to 150 mM NaCl, with substantial unwinding (∼50%) at 200 mM NaCl (Fig. 3a,b). These results demonstrate that WRN unwinds three-way junctions with G-rich telomeric, single-stranded flaps much better than those containing C-rich telomeric flaps, a preference that becomes particularly pronounced at 100–200 mM NaCl concentration. Further, WRN unwinding of *Mobile-G substrate appears more ‘salt resistant’ compared with static three-way junction substrates containing random sequences.

Importantly, these results were our first indication that WRN possessed sequence preferences pertinent to telomere metabolism. However, *Mobile-G substrate was inappropriate for determining the precise positioning of G-rich sequences that mediated WRN’s heightened activity. On the basis of the 3′–5′ directionality of WRN helicase and predominant displacement of 3′ flap strands of three-way junction substrates, we anticipated that G-rich sequences on the 3′ flap strand might facilitate this preferential unwinding. Surprisingly, this was not the case, as three static three-way junction substrates (*3-way asymm, *3-way 3′G3.5X or *3-way 3′C3.5X) containing scrambled G-telomeric, G-telomeric or C-telomeric sequence, respectively, on their 21-nt 3′ flaps were unwound similarly with the expected directionality by WRN across NaCl concentrations (Supplementary Fig. 2a,b). We then constructed another series of static three-way junction substrates, placing comparative sequences of interest on the 5′ flap. Specifically, the 21-nt 5′ flaps of four three-way junction substrates (*3-way jct, *3-way 5′G3.5X, *3-way 5′S3.5X or *3-way 5′C3.5X) contained random, G-telomeric, scrambled G-telomeric or C-telomeric sequences, respectively (Fig. 3c, top), while their 3′ flaps contained an identical scrambled G-telomeric sequence. Here and subsequently, WRN unwinding was measured at ⩾50 mM NaCl, conditions exclusively yielding 3′-flap strand displacement (Fig. 3c), as expected from previous experiments. Importantly, WRN more efficiently unwound the three-way junction substrate with a G-rich telomeric sequence on the 5′ flap compared with substrates with other sequences at this position; this preference was most pronounced at 100–150 mM NaCl (Fig. 3c,d). Interestingly, C-telomeric sequence on the 5′ flap decreased unwinding even when compared with scrambled G-telomeric and random sequences (Fig. 3d). Kinetic experiments (at 150 mM NaCl) confirmed this pattern, at each time point showing significantly higher unwinding of 3-way 5′G3.5X substrate with a G-telomeric sequence on the 5′ flap (Fig. 3e). Unwinding observed on this and other substrates is specifically performed by WRN, as helicase-dead mutant WRN-K577M^31^,^43^ does not detectably unwind 3-way 5′G3.5X substrate (Supplementary Fig. 2c). Collectively, these results indicate that G-rich telomeric sequences (compared with C-rich telomeric and non-telomeric sequences) specifically on the 5′ flap strand of three-way junction substrates confer heightened 3′-flap strand unwinding by WRN. Thus, not only is the 5′ (invading) flap an important determinant of WRN function on strand invasion structures, but its sequence composition also influences WRN’s unwinding strength.

These results suggested that WRN might have enhanced binding affinity towards strand invasion intermediates containing invading strand G-rich telomeric sequences. Indeed, WRN bound a significantly higher percentage of the 3-way 5′G3.5X substrate with the G-rich 5′ flap than 3-way 5′C3.5X with a C-rich 5′ flap (Fig. 3f), consistent with WRN unwinding preference between these substrates (Fig. 3c–e). To further determine whether G-rich telomeric sequence conferred enhanced WRN-binding affinity in a similar structural context, we compared a series of 5′ flap substrates (*5′ Flap, *5′ Flap G3.5X, *5′ Flap S3.5X or *5’ Flap C3.5X) containing random, G-rich telomeric, scrambled G-telomeric or C-telomeric sequences, respectively (Supplementary Fig. 3a, top). Collectively, these substrates were more weakly bound by WRN than three-way junction structures (see Fig. 1e,f), but *5′ Flap G3.5X substrate with G-rich telomeric 5′ flap was more efficiently bound across WRN concentration than other substrates (Supplementary Fig. 3a,b). These results indicate that the presence of G-rich telomeric sequences on 5′ flap regions mediates increased WRN binding. Together with previous results, we deduce that enhanced affinity for G-rich telomeric sequences in specific structural contexts contributes to WRN’s increased and salt-resistant unwinding of strand invasion intermediates with invading strand G-rich telomeric sequences.

**Figure 4:**
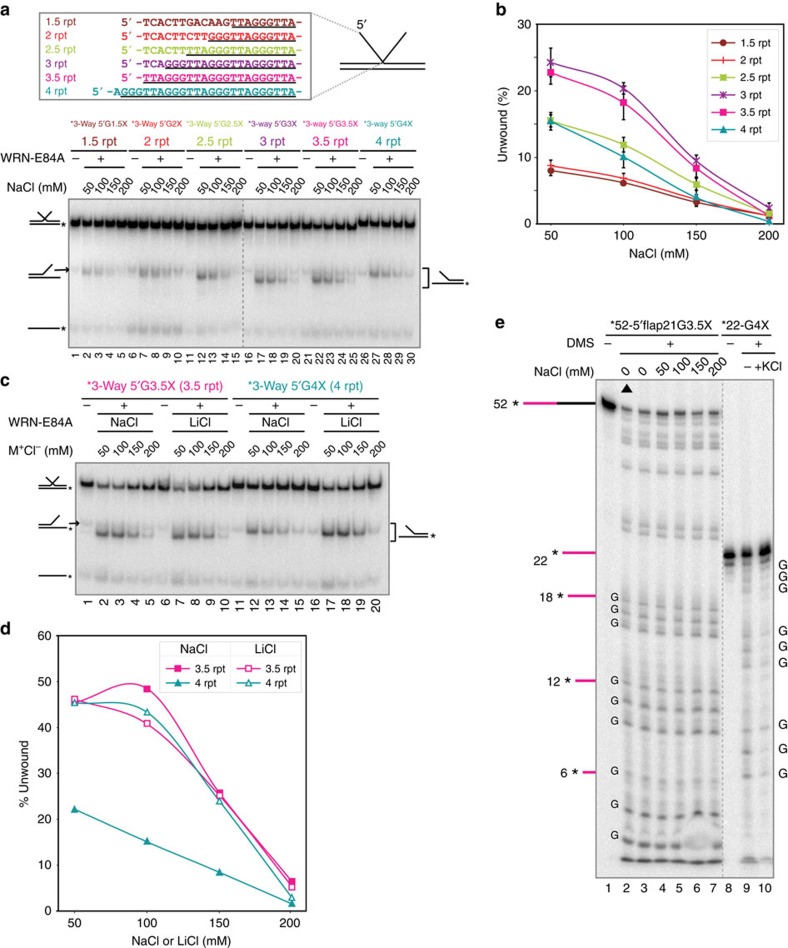
WRN unwinding of strand invasion intermediates is enhanced by ⩾3 unstructured G-rich telomeric repeats on the invading strand. (**a**) Helicase assays performed with WRN-E84A (0.23 nM) incubated for 15 min with *3-way 5′G1.5X (1.5 repeats), *3-way 5′G2X (2 repeats), *3-way 5′G2.5X (2.5 repeats), *3-way 5′G3X (3 repeats), *3-way 5′G3.5X (3.5 repeats) or *3-way 5′G4X (4 repeats) substrate (0.2 nM each) in 50–200 mM NaCl as indicated. Migration positions of substrates and products are denoted. 5′ flap sequences for substrates are shown (box), colour-coded in relation to **a**–**d** with G-rich telomeric sequence underlined. (**b**) Percent unwinding (mean±s.e.m. of three experiments, as in **a**). Asterisks indicate significant differences (*P*<0.04, calculated using two-tailed unpaired Student’s *t*-tests) of data points compared with analogous data points from each other substrate. (**c**) Helicase assays performed with WRN-E84A (0.27 nM) incubated for 15 min with *3-way 5′G3.5X (lanes 1–10) or *3-way 5′G4X (lanes 11–20) substrate (0.2 nM each) in 50–200 mM NaCl or LiCl as specified. Migrations of substrates and products are indicated. (**d**) Percent unwinding from each lane in **c**. (**e**) DMS protection assays were performed on *52-5′flap21G3.5X oligomer (0.2 nM) in 0–200 mM NaCl as specified and *22-G4X oligomer (0.2 nM) in 0 or 75 mM KCl. After denaturing PAGE, uncleaved oligos and fragments resulting from cleavage at DMS-methylated (unprotected) guanines are visualized; solid triangle (lane 2) indicates reaction was heat-denatured before DMS treatment. Fragments ending in guanines within telomeric repeats are denoted with ‘G’; for reference, lengths of substrates and key DNA fragments (G-rich telomeric content in magenta) are indicated at the left.

Because G-rich telomeric sequences can form G-quadruplex structures, particularly in the presence of Na^+^ or K^+^ cations^44^,^45^,^46^,^47^, and WRN can disrupt G-quadruplexes^35^,^48^, we considered the possibility that G-quadruplexes might mediate WRN’s enhanced activity on three-way junction substrates with 5′ flaps containing a G-rich telomeric sequence. First, it was relevant to determine the amount of G-rich telomeric sequence on the 5′ flap required for optimal WRN unwinding. Yet another series of three-way junction substrates (*3-way 5′G1.5X, *3-way 5′G2X, *3-way 5′G2.5X, *3-way 5′G3X, *3-way 5′G3.5X and *3-way 5′G4X) was designed, containing 5′ flaps with 1.5–4 G-telomeric repeats adjacent to the junction; where applicable, their distal 5′ ends had random sequences used previously (Fig. 4a, top). These substrates had 21-nt 5′ flaps except *3-way 5′G4X substrate, its four telomeric repeats necessitating a longer (25 nt) 5′ flap (Fig. 4a, box). Unwinding assays demonstrated that WRN most efficiently acted on *3-way 5′G3X and *3-way 5′G3.5X substrates, while the presence of fewer than three GGG runs on the 5′ flap decreased WRN unwinding (Fig. 4a,b). This preference was consistent and significant across NaCl concentration, except at 200 mM NaCl where overall unwinding was nearly abolished (Fig. 4b). Surprisingly, *3-way 5′G4X substrate containing four G-rich telomeric repeats on the 5′ flap also showed reduced unwinding compared with optimal substrates with 3 or 3.5 repeats (Fig. 4b). Notably, single-stranded DNA containing four telomeric (GGGTTA) repeats forms intramolecular G-quadruplexes in NaCl solutions^47^,^49^, suggesting that intramolecular G-quadruplex formation within this particular substrate might be responsible for its reduced unwinding. Therefore, unwinding of *3-way 5′G3.5X and *3-way 5′G4X substrates with 3.5 and 4 repeats, respectively, was analysed in LiCl, which disfavours G-quadruplex formation^45^,^50^, versus NaCl. As above, *3-way 5′G4X substrate showed markedly lower unwinding than *3-way jct 5′G3.5X from 50 to 150 mM NaCl (Fig. 4c,d). In contrast, WRN activity on these substrates with 3.5 or 4 G-rich repeats was essentially identical in 50–150 mM LiCl and similar to unwinding levels on *3-way jct 5′G3.5X substrate in NaCl (Fig. 4c,d). These results indicate that intramolecular G-quadruplex formation on these 5′ flaps actually decreases WRN-mediated unwinding of three-way junctions.

While results above suggested enhanced WRN unwinding caused by G-rich telomeric sequences was not due to G-quadruplex formation, it remained possible that G-rich strands from multiple substrate molecules could associate to form intermolecular G-quadruplexes. To examine this possibility, the dimethyl sulfate (DMS) protection assay was performed on *52-5′flap21G3.5X oligomer (containing 3.5 single-stranded G-rich telomeric repeats) used to construct *3-way 5′G3.5X substrate, under NaCl and DNA concentration conditions mimicking helicase assays. Guanines not involved in G-quadruplexes are methylated by DMS and thus subject to piperidine cleavage^51^, generating a pattern of DNA fragments after denaturing PAGE^52^. As a positive control for the assay and G-quadruplex formation, we also examined *22-G4X, a 22-mer comprised of G-rich telomeric repeats including four GGG runs (Fig. 4e, lanes 8–10). Without salt, *22-G4X produced a fragment pattern corresponding to cleavage at guanines, indicating a relatively unstructured conformation. Stabilization of G-quadruplexes using 75 mM KCl substantially reduced the intensity of these bands, indicating protection of these guanines by their involvement in G-quadruplexes. In contrast, fragment patterns with 52-5′flap21G3.5X strand over 0–200 mM NaCl (Fig. 4e, lanes 3–7) were essentially identical to a heat-denatured control (lane 2). These results indicate that, under helicase assay conditions, single-stranded G-rich telomeric repeats containing three GGG runs do not detectably form G-quadruplexes. Together, our findings indicate that an unfolded state of the 5′ flap containing at least three G-rich telomeric repeats is mediating both heightened and salt-resistant unwinding of three-way junction substrates by WRN. Hence, our results reveal that WRN has a marked preference for strand invasion structures containing (unstructured) single-stranded G-rich telomeric repeats on the invading strand, exactly mirroring their positioning within T-loops and telomeric HR intermediates.

### Involvement of WRN’s C terminus in DNA substrate specificity

**Figure 5:**
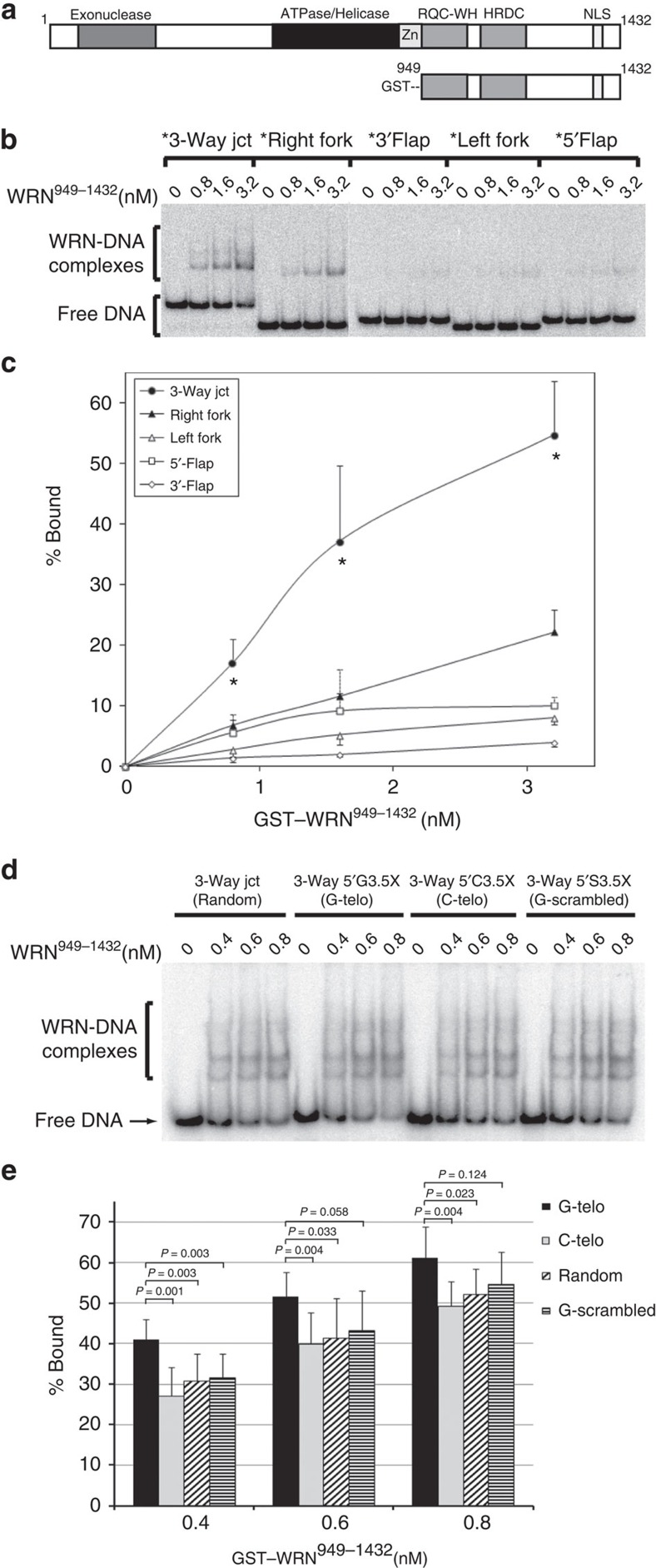
Structure and sequence preferences are conferred by WRN’s C-terminal region. (**a**) Diagram of key domains in full-length WRN and GST–WRN^949–1432^ proteins, showing approximate positions of exonuclease, ATPase/helicase, Zn-finger (Zn), RecQ-conserved-winged helix (RQC-WH), helicase and RNase D-conserved (HRDC) and nuclear localization signal (NLS) domains. (**b**) EMSA performed in 100 mM NaCl without ATPγS on GST–WRN^949–1432^ (0–3.2 nM) incubated for 15 min at 37 °C with *3-way jct, *Right Fork, *Left Fork, *3′ Flap or *5′ Flap substrate (0.1 nM each) as indicated. After adding one-sixth volume of 30% glycerol containing 0.05% BPB, samples were run on native PAGE at 25 °C. (**c**) Percent substrate (*3-way jct, closed circles; *Right Fork, closed triangles; *Left Fork, open triangles; *5′ Flap, open squares; *3′ Flap, open diamonds) bound (mean±s.e.m. of three or four experiments, as in **b**) plotted versus GST–WRN^949–1432^ concentration. Asterisks below data points denote significant differences (*P*<0.001, determined by a two-tailed unpaired Student’s *t*-test) compared with analogous data points from all other substrates. (**d**) Binding reactions in 100 mM NaCl with GST–WRN^949–1432^ (0–0.8 nM) incubated for 10 min at 4 °C with *3-way jct (Random), *3-way 5′G3.5X (Gtelo), *3-way 5′C3.5X (Ctelo) or *3-way 5′S3.5X (Gscr) substrates (0.1 nM each) as specified were analysed as in **b**, except one-sixth volume of 30% glycerol was added before PAGE was performed at 4 °C. (**e**) Bar plot of percent substrate bound (mean±s.e.m. of nine experiments, as in **d**) for each substrate (Gtelo, black; Ctelo, gray; Random, diagonal striped; Gscr, horizontal striped) at each WRN concentration. Statistical significance (*P* values calculated using two-tailed unpaired Student’s *t*-tests) between data points demarcated by brackets is provided. Multiple protein–DNA complexes and variations in their patterns may be due to binding of multiple molecules of GST–WRN^949–1432^ and to differences in their stability under differing electrophoresis conditions.

WRN contains multiple DNA-binding domains, including the RecQ-conserved, winged helix (RQC-WH) and helicase and RNase D-conserved (HRDC) domains in its C-terminal region^53^. To determine whether this region contributed to our observed structure and sequence specificity, we generated a fusion protein (glutathione S-transferase (GST)–WRN^949–1432^) of WRN’s C-terminal region (amino acids 949–1432) including both RQC-WH and HRDC domains, but lacking N-terminal sequences including immediately upstream ATPase–helicase and zinc-finger domains (Fig. 5a). We then used EMSA to examine the structure and sequence specificity of GST–WRN^949–1432^. When comparing various DNA structures without telomeric sequences (Fig. 1a), GST–WRN^949–1432^ clearly bound with highest affinity to basic *3-way jct substrate (Fig. 5b), similar to full-length WRN (compare Fig. 5c with Fig. 1e–g). A GST-only control did not detectably bind DNA (Supplementary Fig. 4). We then compared GST–WRN^949–1432^ binding to three-way junction substrates (*3-way jct, *3-way 5′G3.5X, *3-way 5′C3.5X and *3-way 5′S3.5X) with random, G-rich telomeric, C-rich telomeric and G-scrambled sequences, respectively, on their 5′ flaps (see Fig. 3c). This analysis (Fig. 5d,e) showed, across all concentrations tested, a consistent trend (that reached statistical significance for most pairwise comparisons) for preferential binding of GST–WRN^949–1432^ to *3-way 5′G3.5X substrate containing a G-rich telomeric (Gtelo) sequence on the 5′ flap. When day-to-day variance between experimental repeats (probably due to differential activity of protein aliquots) was taken into account, this observed preference of GST–WRN^949–1432^ was statistically significant when comparing all data points. Notably, the largest and most significant differences in GST–WRN^949–1432^ binding were between substrates containing G-rich versus C-rich telomeric 5′ flap sequences (Fig. 5e), exactly mirroring helicase and binding results using full-length WRN (Fig. 3). These results demonstrate that WRN’s C-terminal region containing the RQC-WH and HRDC DNA-binding domains contributes substantially to heightened specificity for three-way junction structures with G-rich telomeric sequences on their 5′ flap (invading) strands, although involvement of other domains remains possible. Regardless, WRN’s C-terminal region appears to provide considerable structure and sequence specificity to WRN’s helicase activity and to its role in telomere metabolism.

## Discussion

WRN deficiency results in telomeric abnormalities, premature cellular senescence and accelerated development of certain ageing features. Here, we identified a potential role of WRN in processing recombination intermediates formed during telomeric HR events and at T-loops. Our results indicate that WRN unwinds strand invasion intermediates more efficiently than other related DNA structures. Importantly, WRN also possesses sequence specificity consistent with its putative role in telomere maintenance, as its unwinding activity is further enhanced by the presence of a G-rich telomeric sequence specifically on the invading strand (5′ flap) of strand invasion intermediates, analogous to G-rich strand orientation within T-loops and other telomeric HR intermediates. The directionality of WRN unwinding of these structures favours further strand invasion at telomeres, suggesting that WRN might promote proper telomeric HR and potentially assist formation and stabilization of T-loops. Notably, WRN’s definitive unwinding directionality as well as its optimal structure and telomeric sequence discrimination occurs when Na^+^ concentration is 100–150 mM; the effect of ionic concentration on these activities is particularly relevant, considering the reported monovalent cation concentration range (125–250 mM) within the nucleus^40^,^41^,^42^.

In examining WRN’s action on substrates containing random sequences, unwinding of three-way junction/strand invasion substrate was clearly enhanced compared with 5′ flap, 3′ flap and two distinct two-stranded fork substrates, the latter previously shown to be excellent WRN substrates^35^,^54^. Importantly, WRN unwinding of three-way junction structures remained robust at 100 mM NaCl and persisted up to 150 mM NaCl, while unwinding of other substrates was drastically diminished or absent under these conditions. Moreover, complementarity on 5′ flaps (as occurs during HR events) further enhanced WRN unwinding and its salt resistance on three-way junction structures. EMSA studies revealed that this unwinding preference likely reflected enhanced (and perhaps more stable) DNA binding by WRN. Regarding the directionality of WRN unwinding on strand invasion intermediates, displacement of the 3′ flap strand increasingly predominated as NaCl concentration increased. Preferential unwinding of the 3′ flap strand was not unexpected, since 3′–5′ helicases such as WRN characteristically translocate in a 3′–5′ manner along single-stranded DNA to unwind adjacent duplex regions. However, it was surprising that highly efficient and salt-resistant displacement of this 3′ flap strand required the 5′ flap, revealing the importance of this structural feature for WRN’s preference for strand invasion intermediates and foreshadowing our findings with telomeric three-way junction substrates (discussed below). This indicates that WRN senses other structural features of DNA substrates besides the strand along which it translocates; on strand invasion intermediates, the presence of the 5′ flap (invading) strand mediates enhanced and salt-resistant unwinding by WRN. Together, these results strongly support the concept that WRN preferentially targets strand invasion intermediates *in vivo*, acting to promote additional strand invasion and exchange.

Prompted by WRN’s putative but unknown function in telomere metabolism, subsequent experiments revealed WRN’s striking preference (and highly increased salt tolerance) for unwinding partly mobile, three-way junction substrates with single-stranded flaps containing G-rich as opposed to C-rich telomeric sequences. These results indicated that WRN possessed a heretofore unknown sequence preference within the context of three-way junction structures that we further defined by placing different sequences on either the 3′ or 5′ flaps of static three-way junction substrates. Surprisingly, nucleotide sequence on the 5′ flap clearly influenced WRN unwinding, while different 3′ flap sequences had no effect. Precisely, three-way junction substrates with at least three G-telomeric repeats ((GGGTTA)_3_) proximal to the junction on the 5′ flap were optimally unwound when compared with similar substrates with C-telomeric or non-telomeric sequences. Notably, these sequence preferences within the context of three-way junction structures by WRN were maintained in buffers containing K^+^ (data not shown), the more physiologically abundant monovalent cation^55^. DNA-binding studies further showed that WRN has increased affinity for strand invasion intermediates and single-flap structures containing G-rich telomeric sequences, strongly supporting enhanced specificity of WRN alone on G-rich telomeric sequences, at least in certain structural contexts. While nucleotide sequence also influences DNA binding of budding yeast RecQ homologue Sgs1 (ref. 56), WRN’s enhanced specificity for G-rich telomeric sequences is striking, considering its proposed role in telomere maintenance. This observed WRN specificity for substrates with G-rich telomeric sequences suggested possible involvement of G-quadruplex structures, particularly since WRN disrupts G-quadruplexes *in vitro*^35^,^48^. However, our results clearly show otherwise, because (1) ‘optimal’ substrates with three G-rich telomeric repeats do not form these structures at DNA concentrations used in our assays and (2) G-quadruplex formation in this structural context within substrates containing four repeats actually reduces WRN-mediated unwinding. In the latter case, the four G-rich repeats form intramolecular G-quadruplexes that likely conceal the single-stranded 5′ flap, resulting in a poorer WRN substrate. Thus, heightened and optimal WRN unwinding of strand invasion substrates occurs when the (invading) 5′ flap contains at least three G-rich telomeric repeats proximal to the junction point and retains its primary (unfolded) structure. Remarkably, WRN senses this telomeric nucleotide sequence context on the invading strand of strand invasion intermediates while displacing the non-invading strand. Furthermore, a C-terminal WRN fragment lacking the entire ATPase/helicase domain but containing RQC-WH and HRDC domains possesses DNA-binding affinities consistent with binding and unwinding preferences of full-length WRN. This indicates that WRN’s C-terminal region helps confer heightened specificity for both strand invasion intermediates and the G-rich telomeric sequence specifically on the invading strand, although our experiments cannot exclude involvement of its helicase domain or other regions. As earlier reports implicated the RQC-WH domain in binding 5′ single-stranded elements within other DNA structures^53^,^57^, this specific domain probably helps mediate enhanced binding and activity on strand invasion intermediates.

Our findings have major implications for telomere metabolism and understanding previously reported telomere-related abnormalities caused by WRN deficiency. WRN’s high affinity, salt resistance and mechanism of action on strand invasion intermediates with G-rich telomeric sequences specifically on the invading strand theoretically implicate it in at least two telomeric HR processes that help maintain telomere stability. First, WRN may assist in T-loop formation or stabilization. Second, WRN may be involved in promoting normal HR processes within telomeric regions, perhaps in re-establishing replication forks that have collapsed within telomeric regions, via an HR process analogous to break-induced replication pathways^58^. Both processes require (1) strand invasion by G-rich strand 3′ overhangs that occur naturally at telomeric ends or are generated by 5′ strand resection associated with HR and then (2) further exchange of telomeric strands. Importantly, the obligate positioning of G-rich telomeric strands during these strand invasion processes places them in the exact context for which we observe WRN’s most enhanced and salt-resistant activity. The stochastic telomere losses and aberrant telomeric recombination phenotypes associated with WRN deficiency^19^,^20^,^21^,^22^,^29^,^30^,^59^,^60^, possibly due to inability to properly carry out telomeric HR, restore telomeric replication or form stable, protected T-loops that resist nucleolytic degradation, are also consistent with these roles. Telomere loss primarily associated with lagging-strand replication in cells lacking WRN function^20^ might be attributed to the inability to properly initiate or complete an HR process necessary to restart telomeric replication after collapse of lagging-strand replication due to replication blockage occurring (predominantly) at G-quadruplexes and/or DNA damage preferentially formed within telomeric G-rich strands. Notably, telomere-related phenotypes are masked in WRN-deficient cells and mice that possess sufficient telomerase activity^18^,^29^,^30^,^59^, results explained by telomerase-mediated extension of truncated telomeres to restore their normal structure and function.

In regards to its telomeric role, WRN conspicuously interacts physically and functionally with TRF2 and perhaps other telomeric factors^22^,^23^,^24^,^26^,^27^,^28^. TRF2-mediated recruitment of WRN to telomeres likely influences its function and specificity, the latter by essentially eliminating competition from non-telomeric sequences thereby enhancing WRN action upon strand invasion intermediates (or perhaps other structures) with single-stranded G-rich compared with C-rich telomeric sequences. Thus, we speculate that our observed WRN sequence and structure specificity dictates its mechanism of action in telomeric HR and/or T-loop dynamics, aided by its recruitment to telomeres or specific telomeric structures by TRF2 alone or in association with other shelterin components. These concepts are strongly supported by our earlier finding that TRF2 and WRN cooperate to catalyse strand exchange specifically between telomeric DNAs^28^. During such strand-exchange reactions, TRF2- and WRN-generated strand invasion intermediates are processed further by the latter’s helicase activity, actions presumably enhanced through WRN’s structure and telomeric sequence preferences shown here. Importantly, TRF2 is an essential factor in telomeric end protection^7^,^61^,^62^,^63^, putatively through its involvement in T-loop formation and stabilization^10^,^11^,^12^ and its preferential binding at the junction between telomeric duplexes and G-rich 3′ overhangs^11^. Fittingly, WRN also readily acts on four-stranded strand invasion intermediates containing a telomeric duplex sequence specifically on the invading arm, even in the presence of a 10-fold molar excess of TRF2 (Supplementary Fig. 5). Together, this evidence strongly supports the concept that WRN acts (with TRF2) in T-loop dynamics and/or other telomeric HR processes, although additional research is needed to clarify this further. Regardless, our findings bolster the concept that telomere dysfunction is involved in manifestation of ageing phenotypes, at least in WS if not also in normal ageing.

## Methods

### Enzymes

WRN-E84A has a point mutation that eliminates its exonuclease activity^32^ but preserves its 3′–5′ helicase and annealing activities. WRN-K577M contains a point mutation that eliminates its ATPase and helicase activities^31^,^43^. Using appropriate *WRN* cDNA sequences cloned into baculoviral constructs, WRN-E84A and WRN-K577M were overexpressed in *Sf9* insect cells and purified after cell lysis by consecutive DEAE-Sepharose, Q-Sepharose and NiNTA Agarose liquid chromatography steps performed at 4 °C (ref. 64) with inclusion of 0.1% Nonidet P40 in all liquid chromatography buffers. Protein aliquots were collected, supplemented with 100 μg ml^−1^ bovine serum albumin, flash frozen and stored at −80 °C until use. Using standard methods (GE Healthcare), pGEX plasmids carrying GST and GST–WRN^949–1432^ fragments were independently transformed into competent *Escherichia coli* (BLR strain); the corresponding proteins were overexpressed by growth for 8 h at 24 °C after induction using isopropylthiogalactose (1 mM) and purified after lysis using Glutathione Sepharose 4B affinity resin. DNase I, and T4 polynucleotide kinase were purchased from New England Biolabs (Ipswich, MA).

### DNA substrates

All oligonucleotides (see Supplementary Table 1 for nucleotide sequences) were purchased from Integrated DNA Technologies (Coralville, IA) and PAGE-purified. The oligos 62-base, 83-Mobile3-G3.5X, 52-5′flap21G3.5X or 22-G4X were 5′ radiolabelled using T4 polynucleotide kinase and ^32^P-γ-ATP, and reactions were passed through Mini Quick Spin Oligo Columns (Roche, Indianapolis, IN) to remove unincorporated ATP; elsewhere, labelled strands and substrates are indicated by asterisks. To form most three-way junction, single-flap and two-stranded fork substrates, excess amounts of appropriate unlabelled oligonucleotides (5′-flap and/or 3′-flap strand variations, or Mobile three-way junction oligos) were annealed to *62-base or *83-Mobile3-G3.5X (see Supplementary Table 2 for substrate names and oligo composition) in 50 mM Tris (pH 8.0) and 10 mM MgCl_2_ by heating to 90 °C followed by step-wise slow cooling (decreasing temperature in 5 °C increments, held for 5 min each). In contrast, three-way junction substrates with partly complementary flaps, *3-way 5′comp or *3-way 3′comp, were generated by first annealing an excess of 52-3′flap21S3.5X or 52-5′flap21R, respectively, to *62-base as described above, followed by annealing a slight excess of 52-5′flap21Rcomp or 52-3′flap21Rcomp to the respective fork at 25 °C overnight. Native PAGE (6%) in 1 × TBE (45 mM Tris pH 8.0, 45 mM boric acid and 1 mM EDTA) was employed to separate unannealed strands from labelled substrates that were then excised and eluted by passive diffusion into 10 mM Tris (pH 8.0) and 10 mM NaCl.

### Helicase assays

Helicase assays were carried out for 0–15 min at 37 °C with WRN-E84A (0.1–3.4 nM) or WRN-K577M (0.2–0.9 nM) in 10 μl WRN reaction buffer (40 mM Tris-HCl (pH 8.0), 1 mM MgCl_2_, 5 mM dithiothreitol, 100 μg ml^−1^ bovine serum albumin, 0.1% NP40 and 250 μM ATP, unless otherwise indicated) with labelled substrate (0.2 nM) and 0–300 mM NaCl (as specified in Results). Reactions were stopped by adding 0–0.16% SDS and 4 mM EDTA and treated with 0.4 mg ml^−1^ proteinase K at 37 °C for 10 min. After adding one-sixth volume of loading dyes (30% glycerol, 50 mM EDTA, 0.25% BPB and 0.25% XC), DNA species were separated by native PAGE (6–8%, specified in figure legends) in 1 × TBE. After gel drying, labelled products were visualized and quantified using a Storm 860 Phosphoimager and ImageQuant software (GE Healthcare). Helicase activity (% unwound) was quantified by comparing the signal of unwinding products to total radioactivity in each lane. Where indicated, significance in helicase and EMSA experiments was calculated using two-tailed unpaired Student’s *t*-tests. Uncropped images of key helicase and EMSA experiments are presented in Supplementary Figs 6 and 7.

### Electrophoretic mobility shift assay (EMSA)

To examine protein–DNA binding, EMSA was performed in 20 μl WRN reaction buffer, except ATP was substituted with 250 μM ATPγS (unless otherwise indicated), along with 50–100 mM NaCl, labelled substrate (0.1–0.2 nM) and WRN-E84A (0.03–3.4 nM), GST–WRN^949–1432^ (0.4–3.2 nM) or GST (0.1–100 nM), incubated at 4 or 37 °C for 10–15 min. Either 1/6 volume of glycerol (30%) or loading dyes as specified was added to these binding reactions and protein–DNA complexes were resolved from unbound substrate by native PAGE (3.5%, 37.5:1) run at 4 °C or 25 °C in 0.5 × TBE without NaCl. Conditions for experiments are specified in the Results and/or the figure legends. Labelled DNA and DNA–protein complexes were visualized by phosporimaging and quantified (using ImageQuant software), with % bound equal to shifted DNA/total DNA radioactivity (× 100) for each lane.

### DNase I footprinting

DNase I footprinting was performed in 10 μl WRN reaction buffer without NaCl. Radiolabelled *3-way jct substrate (2.1 nM) was pre-incubated with WRN-E84A (0–5.8 nM) at 4 °C for 5 min, DNase I (1 U ml^−1^) was added for 10 min at 25 °C and reactions were stopped by adding 10 μl of formamide loading buffer (95% formamide, 20 mM EDTA, 0.05% XC and 0.05% BPB). DNA products were heat-denatured at 90 °C for 5 min and separated by denaturing PAGE (12%). After gel drying, labelled products were visualized by phosphorimaging.

### DMS protection assay

Oligonucleotide *52–5′flap21G3.5X (0.2 nM) in WRN reaction buffer containing 0–200 mM NaCl was treated with 0.5% DMS at 25 °C for 10 min, and reactions were stopped by adding 750 mM β-mercaptoethanol and 1.125 M sodium acetate (pH 7.0). As a control for G-quadruplex formation, *22-G4X was treated as above but in 0 or 75 mM KCl. After adding yeast transfer RNA (10 μg) as carrier, DNA from each sample was collected using ethanol precipitation. Resulting pellets were resuspended in 10% piperidine, then incubated at 90 °C for 30 min and liquid was removed using vacuum evaporation. These samples were dissolved in 10 mM Tris (pH 8.0) and an equal volume of formamide loading buffer was added. To facilitate comparison between samples, equal amounts of radioactivity in individual samples were heat-denatured and analysed by denaturing PAGE (14%) with phosphorimaging.

## Additional information

**How to cite this article:** Edwards, D. N. *et al*. The DNA structure and sequence preferences of WRN underlie its function in telomeric recombination events. *Nat. Commun.* 6:8331 doi: 10.1038/ncomms9331 (2015).

## Supplementary Material

Supplementary Information Supplementary Figures 1-7 and Supplementary Tables 1-2
