# Supplementary material for: The DNA structure and sequence preferences of WRN underlie its function in telomeric recombination events
Source: Nat Commun. 2015 Sep 30;6:8331. doi: 10.1038/ncomms9331 (PMC4589872; doi:10.1038/ncomms9331)
Supplement: Supplementary Information — Supplementary Figures 1-7 and Supplementary Tables 1-2 [file ncomms9331-s1.pdf]

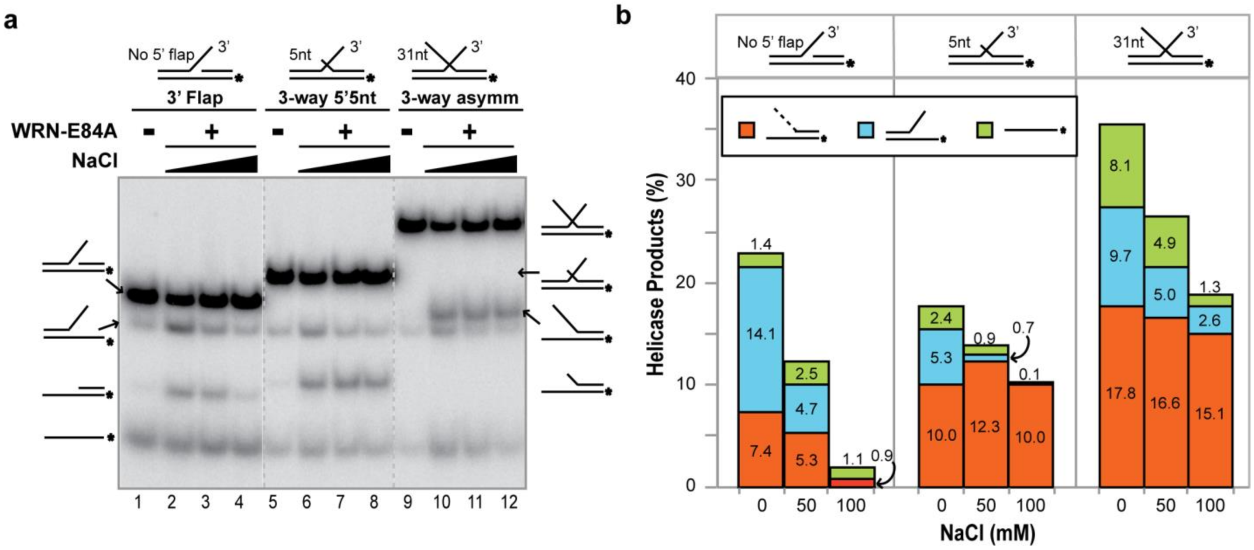

Supplementary Figure 1

Supplementary Figure 1. Single-stranded 5' flap required for enhance unwinding of strand invasion intermediates. a) Helicase assays were performed as in Online Methods on WRN-E84A (0.6 nM) incubated for 15 min in 0, 50, or 100 mM NaCl with *3' Flap (lanes 1-4), *3-way 5'5nt (lanes 5-8), or *3-way asymm (lanes 9-12) substrate (0.2 nM each). Migration positions of substrates and products are denoted. Dotted vertical lines indicate where separated portions of the same gel image were spliced together. **b)** Stacked bar plot showing percent of individual and cumulative products (expressed relative to total substrate amount) generated for each substrate at each NaCl concentration from helicase assay in *(a)*.

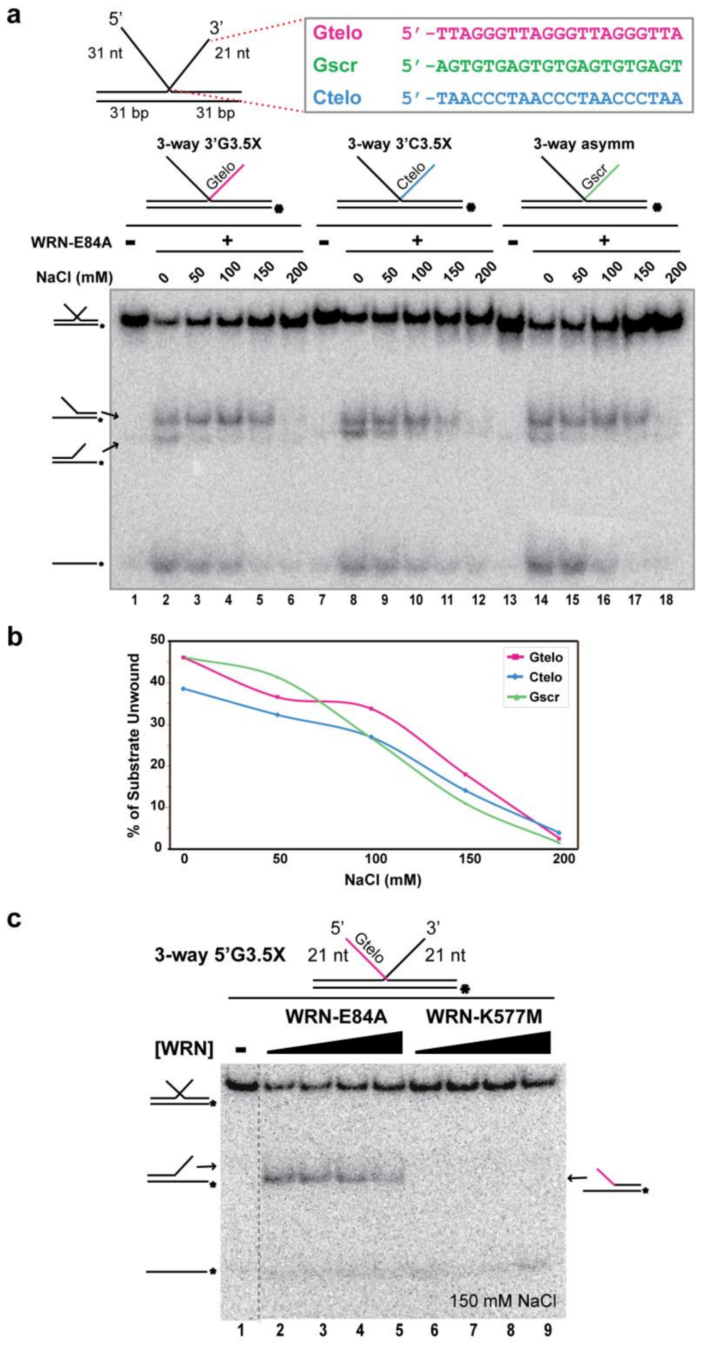

Supplementary Figure 2

Supplementary Figure 2. Telomeric sequence on 3' flap does not affect unwinding that is specifically performed by WRN. a) Helicase assays were performed as in Online Methods on WRN-E84A (0.7 nM) incubated for 15 min with *3-way 3'G3.5X (Gtelo), *3-way 3'C3.5X (Ctelo), or *3-way asymm (Gscr) substrate (0.2 nM each) in 0-200 mM NaCl as specified in figure. Specific sequences of 3' flaps for each substrate are shown (box). Unwinding products were analyzed by native PAGE (6%). Migration positions of substrates and products are indicated. **b)** Percent of substrate unwound (including fork and single-strands products) calculated from *(a)* was plotted versus NaCl concentration for each substrate, using color and 3' flap strand composition coding as in *(a)*.**c)** Helicase assays were performed as in Online Methods in 150 mM NaCl using WRN-E84A (0, 0.22, 0.43, 0.65, or 0.86 nM; lanes 1-5,respectively) or helicase-dead WRN-K577M (0.22, 0.43, 0.65, or 0.86 nM; lanes 6-9,respectively) incubated for 15 min with *3-way 5'G3.5X substrate (0.2 nM). Migration positions of substrate and products are shown. Dotted vertical line indicates where separated portions of the same gel image were spliced together.

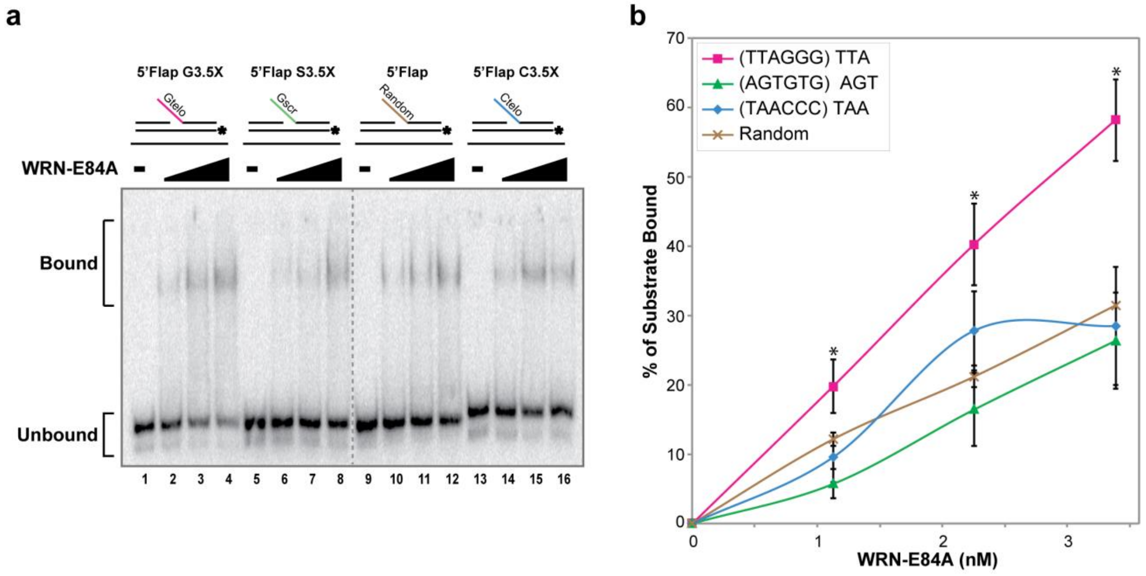

Supplementary Figure 3

Supplementary Figure 3. WRN preferentially binds 5' flap substrates with G-rich telomeric sequence. a) EMSA performed on WRN-E84A (0, 1.1, 2.3, or 3.4 nM) incubated for 10 min at 37°C in 100 mM NaCl with *5' Flap G3.5X (lanes 1-4), *5' Flap S3.5X (lanes 5-8), *5' Flap (lanes 9-12), or *5' Flap C3.5X (lanes 13-16) substrate (0.2 nM each) as described in Online Methods, adding one-sixth volume 30% glycerol containing BPB and XC (0.25% each) before native PAGE at 25°C. Substrate structures shown at top and migration positions of free DNA and WRN-DNA complexes are denoted. Dotted vertical line indicates where separated portions of the same gel image were spliced together. **b)** Percent of substrate bound (mean ± SEM of 5 independent experiments, performed as in *[a]*) is plotted versus WRN concentration. Color coding and labels are consistent with structural diagrams in *(a)*, based on composition of 5' flap region for each substrate. Asterisks denote statistical significance ($p < 0.029$, calculated using two-tailed, unpaired Student's t-tests) of data points compared to analogous data points for each other substrate.

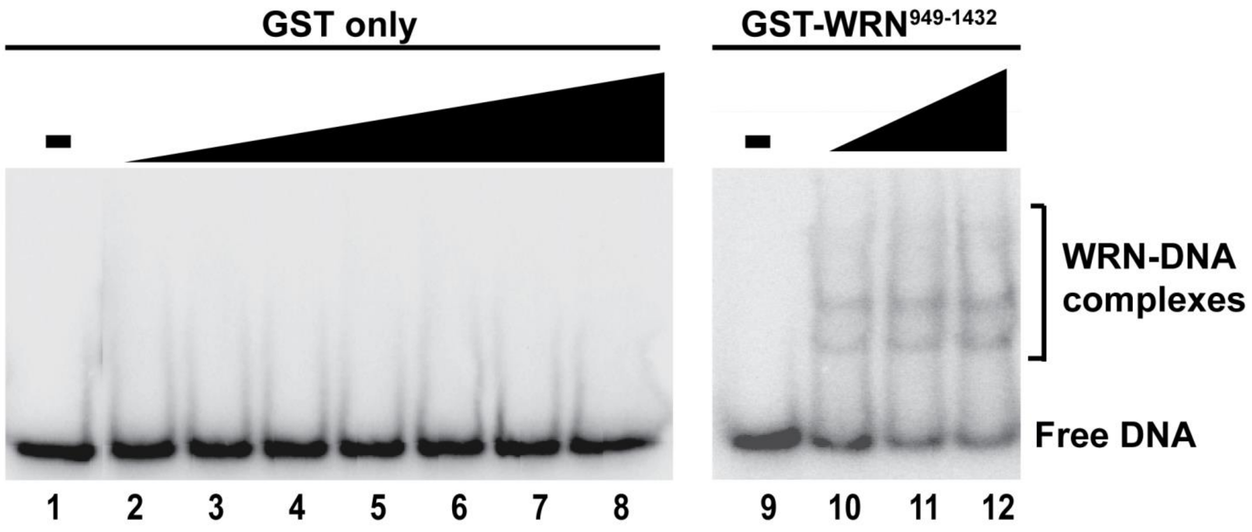

Supplementary Figure 4

Supplementary Figure 4. GST alone does not detectably bind the 3-way junction random substrate. EMSA performed in 100 mM NaCl with *3-way jct substrate (0.1 nM) incubated for 10 min at 4°C with GST (0-100 nM) or GST-WRN$^{949-1092}$ (0-0.8 nM) was analyzed as described in Online Methods, with addition of one-sixth volume of 30% glycerol before native PAGE was run at 4°C. Migration positions of free DNA and WRN-DNA complexes are indicated.

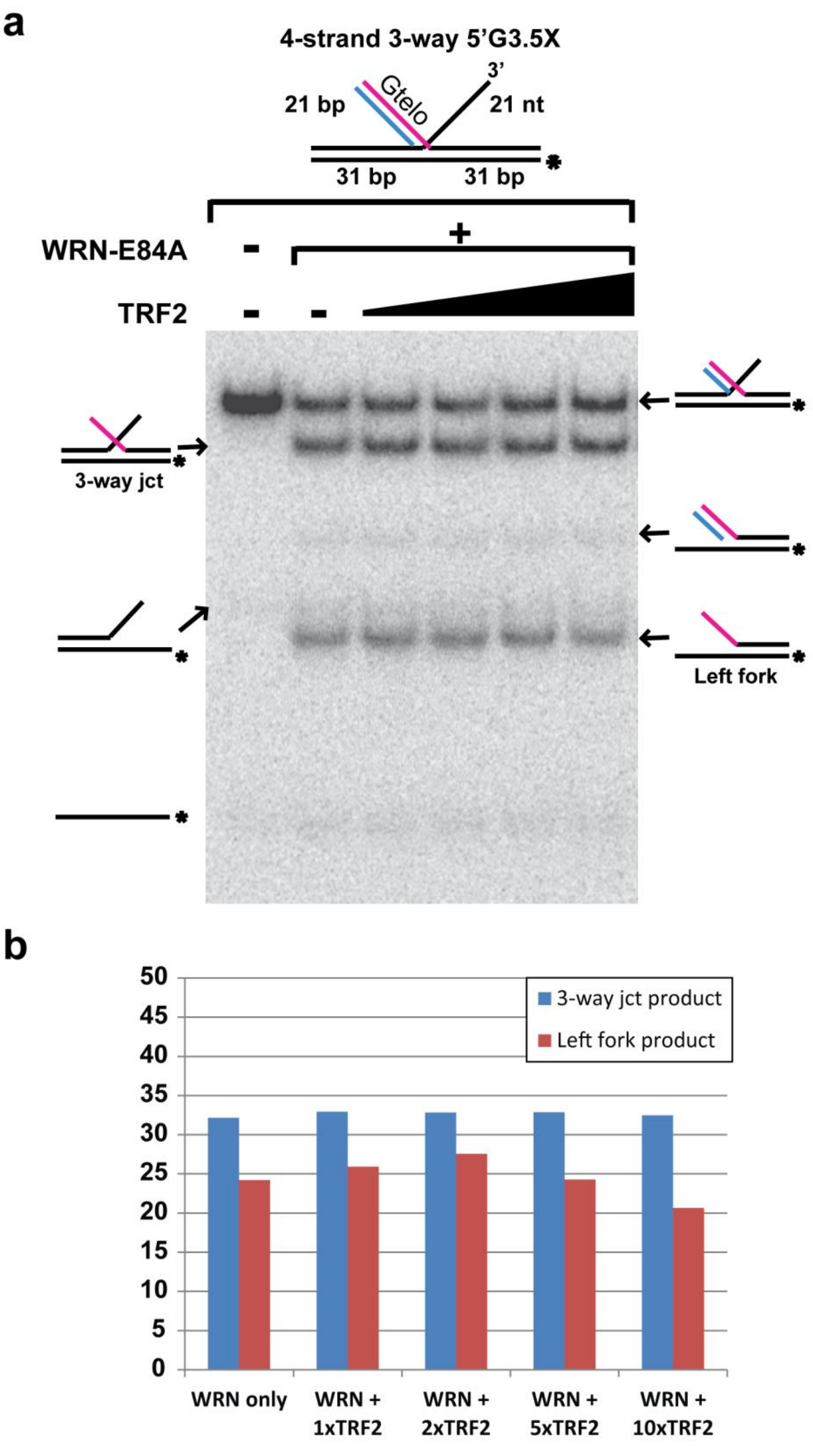

Supplementary Figure 5

Supplementary Figure 5. WRN readily unwinds a telomeric 4-stranded strand invasion intermediate in the presence of TRF2. a) The *4-strand 3-way 5'G3.5X substrate (0.2 nM) was incubated in WRN helicase buffer plus 100 mM NaCl without (lane 2) or with TRF2 (lanes 3-6, 0.325 [1X], 0.65 [2X], 1.625 [5X], 3.25 nM [10X], respectively) followed by WRN-E84A (0.325 nM) where indicated for 5 min at 4°C then at 37°C for 15 min. DNA products were analyzed as described in Online Methods. Positions of substrate and various unwinding products are denoted. **b)** From protein-containing reactions depicted in (*a*), percentages of the primary unwinding products, 3-way junction (*blue*) and 2-stranded Left fork (*red*), were calculated with respect to total DNA signal and plotted in bar graph form. Note that WRN readily generates both unwinding products even in the presence of a 10-fold molar excess of TRF2.

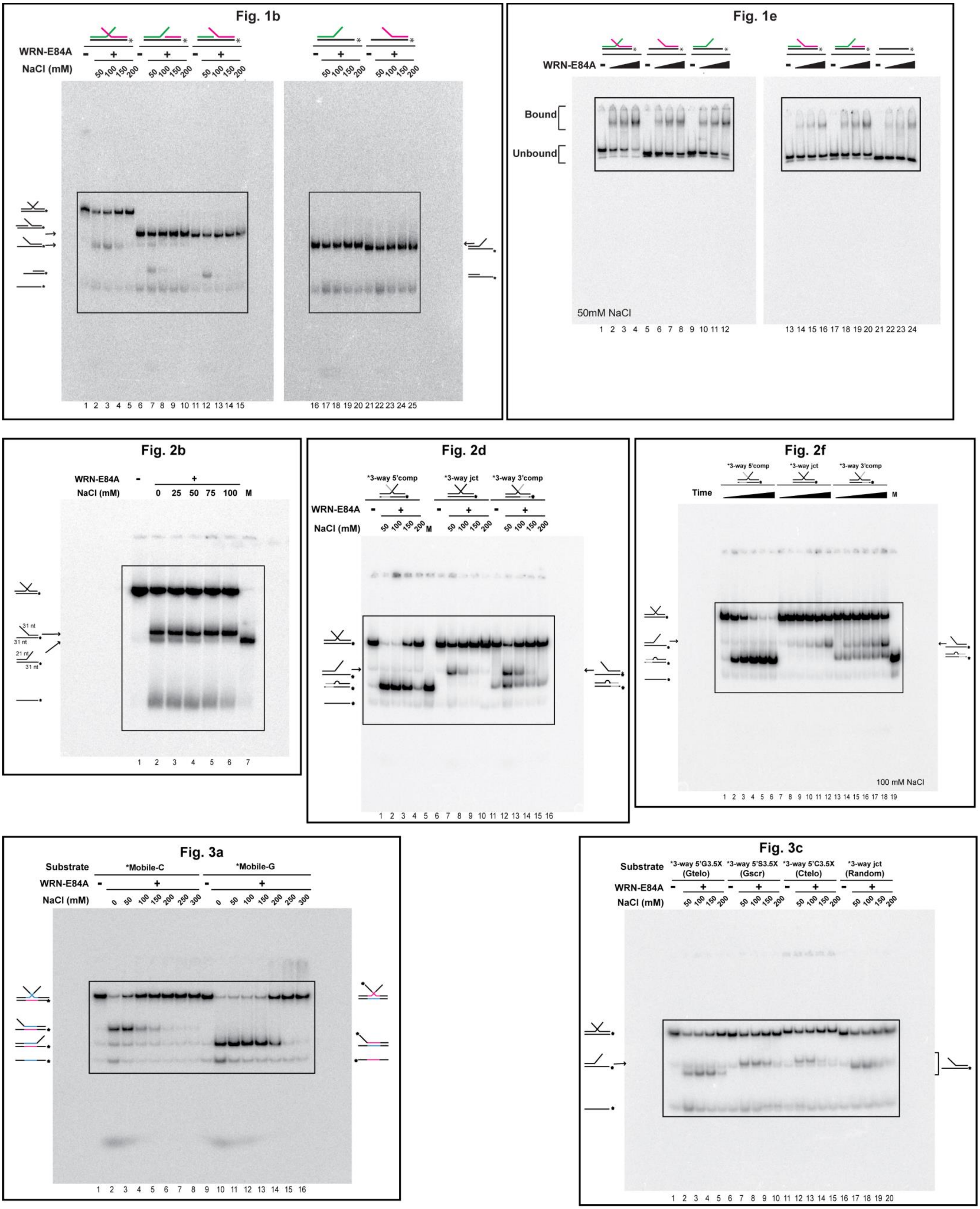

Supplementary Figure 6. Uncropped gel images of key results from figures 1-3.

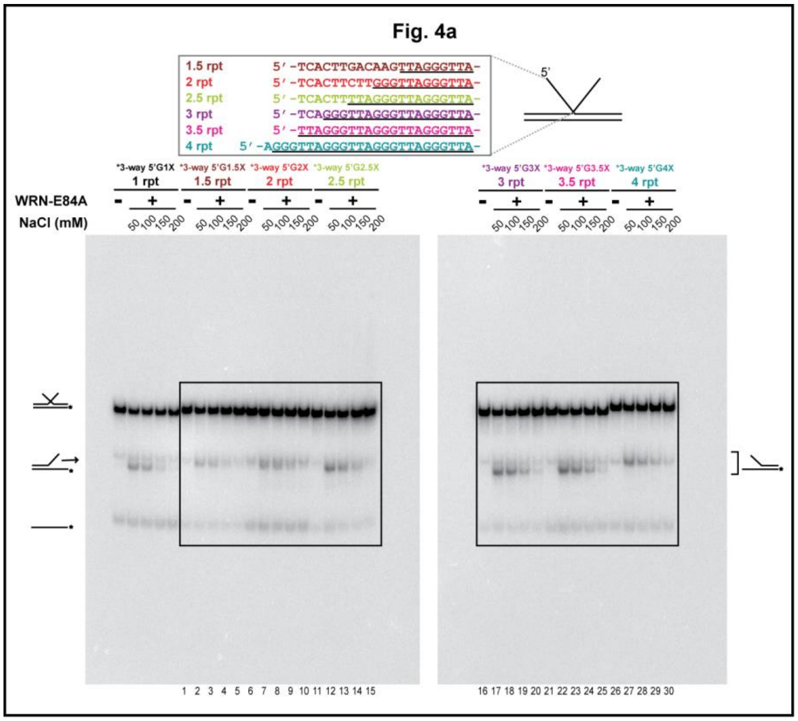

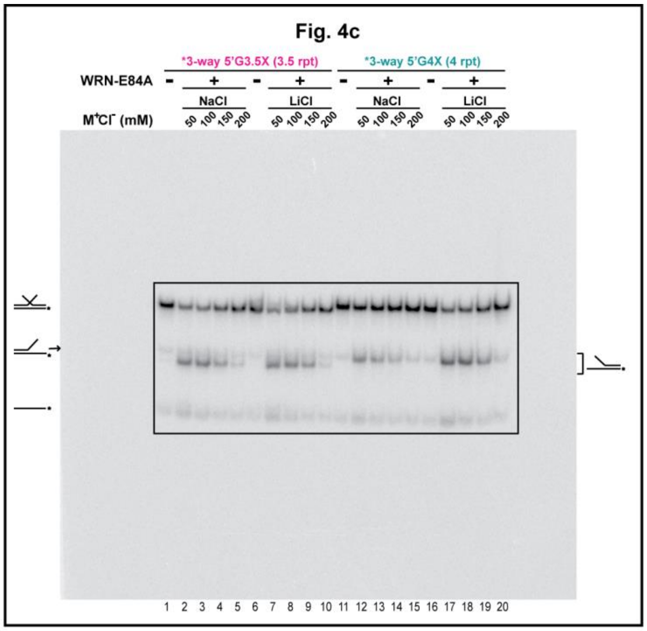

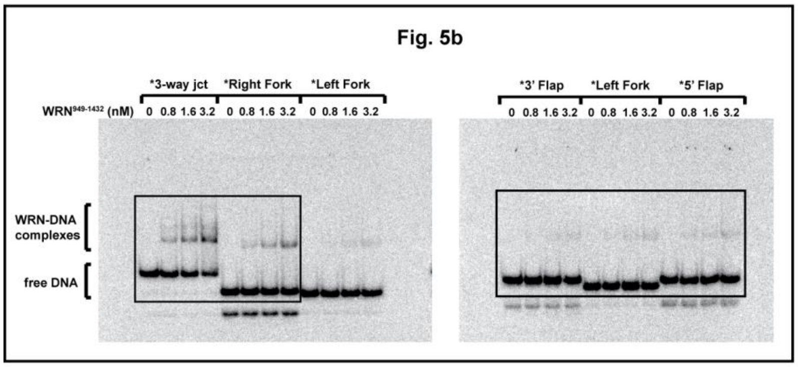

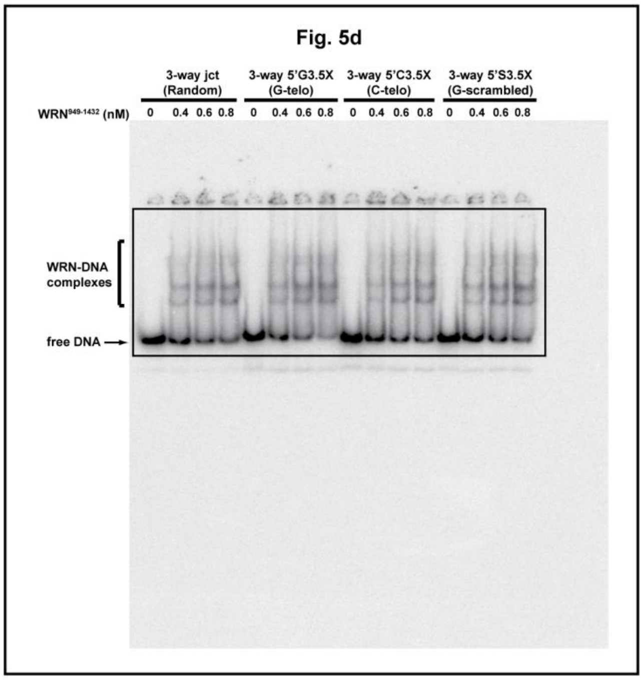

Supplementary Figure 7. Uncropped gel images of key results from figures 4-5.

Supplementary Table 1. Oligonucleotides Used in This Study.

Oligo Name[a]	Nucleotide sequence (in 5' to 3' orientation)[b]
Common Strand for Static 3-way Junction, Single Flap, 2-stranded Fork, and Single-stranded Substrates	
62-base	CACTGACTCCAGGAACTGGAGGATGCCTAGGTGGCCAGCTGCCGTCCAGACTCAGAGGAGTG
5' Flap Strand Variations	
31-5'flap0	CCTAGGCATCCTCCAGTTCCTGGAGTCAGTG
36-5'flap5R	CTAGTCCTAGGCATCCTCCAGTTCCTGGAGTCAGTG
52-5'flap21Rcomp	AGTCTGGACGGCAGCTTGTGACCTAGGCATCCTCCAGTTCCTGGAGTCAGTG
62-5'flap31R	CACTTGGACCTCAAGTCCGACTCAGGCTAGTCCTAGGCATCCTCCAGTTCCTGGAGTCAGTG
52-5'flap21R	TCACTTGACAAGTGACTGTGACCTAGGCATCCTCCAGTTCCTGGAGTCAGTG
52-5'flap21C3.5X	TAACCCTAACCCTAACCCTAACCTAGGCATCCTCCAGTTCCTGGAGTCAGTG
52-5'flap21S3.5X	AGTGTGAGTGTGAGTGTGAGTCCTAGGCATCCTCCAGTTCCTGGAGTCAGTG
52-5'flap21G3.5X	TTAGGGTTAGGGTTAGGGTTACCTAGGCATCCTCCAGTTCCTGGAGTCAGTG
52-5'flap21G3X	TCAGGGTTAGGGTTAGGGTTACCTAGGCATCCTCCAGTTCCTGGAGTCAGTG
52-5'flap21G2.5X	TCACTTTTAGGGTTAGGGTTACCTAGGCATCCTCCAGTTCCTGGAGTCAGTG
52-5'flap21G2X	TCACTTCTTGGGTTAGGGTTACCTAGGCATCCTCCAGTTCCTGGAGTCAGTG
52-5'flap21G1.5X	TCACTTGACAAGTTAGGGTTACCTAGGCATCCTCCAGTTCCTGGAGTCAGTG
56-5'flap25G4X	AGGGTTAGGGTTAGGGTTAGGGTTACCTAGGCATCCTCCAGTTCCTGGAGTCAGTG
3' Flap Strand Variations	
31-3'flap0	CACTCCTCTGAGTCTGGACGGCAGCTGGCCA
52-3'flap21Rcomp	CACTCCTCTGAGTCTGGACGGCAGCTGGCCAAGTGTGCATCCTCCAGTTCCT
52-3'flap21C3.5X	CACTCCTCTGAGTCTGGACGGCAGCTGGCCATAACCCTAACCCTAACCCTAA
52-3'flap21S3.5X	CACTCCTCTGAGTCTGGACGGCAGCTGGCCAAGTGTGAGTGTGAGTGTGAGT
52-3'flap21G3.5X	CACTCCTCTGAGTCTGGACGGCAGCTGGCCATTAGGGTTAGGGTTAGGGTTA
Oligo Added to Create Telomeric 4-stranded 3-way Junction Substrate	
21-C3.5X	TAACCCTAACCCTAACCCTAA
Mobile 3-way Junction Oligos	
83-Mobile1-C3.5X	CACTGGTGACCTGTGCAGAGGCGGAAGGCCTTAACCCTAACCCTAACCCTAAGCTAGCCTGAGTCGGACTTGAGGTCCAAGTG
83-Mobile2-C3.5X	CACTGACTCCAGGAACTGGAGGATGCCTAGGTAACCCTAACCCTAACCCTAATGGCCAGCTGCCGTCCAGACTCAGAGGAGTG
83-Mobile3-G3.5X	CACTCCTCTGAGTCTGGACGGCAGCTGGCCATTAGGGTTAGGGTTAGGGTTAAGGCCTTCCGCCTCTGCACAGGTCACCAGTG
83-Mobile4-G3.5X	CACTTGGACCTCAAGTCCGACTCAGGCTAGCTTAGGGTTAGGGTTAGGGTTACCTAGGCATCCTCCAGTTCCTGGAGTCAGTG
Positive Control for Detection of G-Quadruplex Structure by DMS Assay	
22-G4X	AGGGTTAGGGTTAGGGTTAGGG

[a]Within oligo names, the initial number indicates total nucleotide length while R, C, G, and S signify random sequences, C-telomeric repeats, G-telomeric repeats, and scrambled G-telomeric repeats, respectively, followed by the number of repeats (1-4X) present.
[b]C-telomeric, G-telomeric, and scrambled G-telomeric sequences within oligos are presented in blue, red, and green, respectively.

Supplementary Table 2. Composition of DNA Substrates Used.

Substrate Name	Oligos Used For Substrate Construction[a]			Relevant Figures
	Static 3-way Junction Substrates and Related Structures			
	Common Oligo	3' Flap Strand Variation	5' Flap Strand Variation	
62-base	*62-base	-	-	Fig. 1a,e,f
Right Fork	*62-base	52-3'flap21S3.5X	-	Figs. 1a-g; 5b,c
Left Fork	*62-base	-	52-5'flap21R	Figs. 1a-c,e,f; 5b,c
3' Flap	*62-base	52-3'flap21S3.5X	31-5'flap0	Figs. 1a-f; 5b,c; S. Fig. 1a,b
5' Flap	*62-base	31-3'flap0	52-5'flap21R	Fig. 1a-c,1e,1f; 5b, 5c, S. Fig. 3a,b
3-way jct	*62-base	52-3'flap21S3.5X	52-5'flap21R	Figs. 1a-h; 2d-g; 3c-f; 5b-e; S. Fig. 4
3-way asymm	*62-base	52-3'flap21S3.5X	62-5'flap31R	Fig. 2a-c; S. Figs. 1a,b, 2a,b
3-way 5'comp	*62-base	52-3'flap21S.3.5X	52-5'flap21Rcomp	Fig. 2d-g
3-way 3'comp	*62-base	52-3'flap21Rcomp	52-5'flap21R	Fig. 2d-g
3-way 5'G3.5X[b]	*62-base	52-3'flap21S3.5X	52-5'flap21G3.5X	Fig. 3c-f; 4a-d; 5d,e; S. Figs. 2c, 5a,b
3-way 5'S3.5X	*62-base	52-3'flap21S3.5X	52-5'flap21S3.5X	Fig. 3c-e; 5d,e
3-way 5'C3.5X	*62-base	52-3'flap21S3.5X	52-5'flap21C3.5X	Fig. 3c-f; 5d,e
3-way 5'G1.5X	*62-base	52-3'flap21S3.5X	52-5'flap21G1.5X	Fig. 4a,b
3-way 5'G2X	*62-base	52-3'flap21S3.5X	52-5'flap21G2X	Fig. 4a,b
3-way 5'G2.5X	*62-base	52-3'flap21S3.5X	52-5'flap21G2.5X	Fig. 4a,b
3-way 5'G3X	*62-base	52-3'flap21S3.5X	52-5'flap21G3X	Fig. 4a,b
3-way 5'G4X	*62-base	52-3'flap21S3.5X	52-5'flap21G4X	Fig. 4a-d
3-way 5'5nt	*62-base	52-3'flap21S3.5X	36-5'flap5R	S. Fig. 1a,b
3-way 3'G3.5X	*62-base	52-3'flap21G3.5X	62-5'flap31R	S. Fig. 2a,b
3-way 3'C3.5X	*62-base	52-3'flap21C3.5X	62-5'flap31R	S. Fig. 2a,b
5' Flap G3.5X	*62-base	31-3'flap0	52-5'flap21G3.5X	S. Fig. 3a,b
5' Flap S3.5X	*62-base	31-3'flap0	52-5'flap21S3.5X	S. Fig. 3a,b
5' Flap C3.5X	*62-base	31-3'flap0	52-5'flap21C3.5X	S. Fig. 3a,b
	Mobile 3-way Junction Substrates			
Mobile-C	*83-Mobile3-G3.5X	83-Mobile2-C3.5X	83-Mobile1-C3.5X	Fig. 3a,b
Mobile-G	83-Mobile1-C3.5X	83-Mobile4-G3.5X	*83-Mobile3-G3.5X	Fig. 3a,b

[a]Asterisks indicate radiolabeled oligo for indicated substrates in most applications. Pink shading highlights differences from basic 3-way junction (3-way jct) substrate (yellow shading) containing random 5' flap sequence.
[b]For Supplementary Figure 5 only, oligo 21-C3.5X was annealed to 3-way 5'G3.5X substrate to create *4-strand 3-way 5'G3.5X substrate.
